# Post Mortem Validation of MRI-Identified Veins on the Surface of the Cerebral Cortex as Potential Landmarks for Neurosurgery

**DOI:** 10.3389/fnins.2017.00355

**Published:** 2017-06-21

**Authors:** Günther Grabner, Thomas Haider, Mark Glassner, Alexander Rauscher, Hannes Traxler, Siegfried Trattnig, Simon D. Robinson

**Affiliations:** ^1^Department of Biomedical Imaging and Image-guided Therapy, High Field Magnetic Resonance Centre, Medical University of ViennaVienna, Austria; ^2^Department of Radiologic Technology, Carinthia University of Applied SciencesKlagenfurt, Austria; ^3^Department of Trauma Surgery, Medical University of ViennaVienna, Austria; ^4^Department of Photography, University of Applied ArtsVienna, Austria; ^5^Division of Neurology, Department of Pediatrics, University of British ColumbiaVancouver, BC, Canada; ^6^UBC MRI Research Centre, University of British ColumbiaVancouver, BC, Canada; ^7^Center of Anatomy and Cell Biology, Medical University of ViennaVienna, Austria

**Keywords:** human cadaver, magnetic resonance imaging, post mortem validation, surface vein segmentation

## Abstract

**Background and Objective:** Image-guided neurosurgery uses information from a wide spectrum of methods to inform the neurosurgeon's judgement about which tissue to resect and which to spare. Imaging data are registered to the patient's skull so that they correspond to the intraoperative macro- and microscopic view. The correspondence between imaging and optical systems breaks down during surgery, however, as a result of cerebro-spinal fluid drain age, tissue resection, and gravity-based brain shift. In this work we investigate whether a map of surface veins, automatically segmented from MRI, could serve as additional reference system.

**Methods:** Gradient-echo based ${\text{T}}_{2}^{*}$-weighted imaging was performed on two human cadavers heads using a 7 Tesla MRI scanner. Automatic vessel segmentation was performed using the Frangi vesselness filter, and surface renderings of vessels compared with photographs of the surface of the brain following craniotomy.

**Results:** A high level of correspondence was established between vessel maps and the post autopsy photographs. Corresponding veins, including the prominent superior anastomotic veins, could be identified in all brain lobes.

**Conclusion:** Automatic surface vessel segmentation is feasible and the high correspondence to post autopsy photographs indicates that they could be used as an additional reference system for image-guided neurosurgery in order to maintain the correspondence between imaging and optical systems.This has the advantage over a skull-based reference system that veins are clearly visible to the surgeon and move and deform with the underlying tissue, potentially making this surface net of landmarks robust to brain shift.

## Introduction

Image-guided neurosurgery uses information from an increasingly diverse spectrum of Magnetic Resonance Imaging (MRI) methods to inform the neurosurgeon's judgement about which tissue to resect and which to spare. Anatomical MRI scans with various weightings, T_1_ contrast enhancement and multivoxel spectroscopy allow pathology to be depicted, for instance, while functional MRI activation maps and Diffusion Tensor Images indicate critical populations of neurons and fiber tracts which need to be preserved. Imaging data from these modalities are pair-point registered to the patient's skull via skin-affixed or implanted markers which are visible in both the images and on the head so that they correspond to the intraoperative macro- and microscopic view at the start of the operation (Kubben et al., 2011; Risholm et al., 2011; D'amico et al., 2014). The correspondence between image space and physical space is established by marking the same features or markers in the imaging data (on a computer screen) and in physical space (on the patient) using the tracked pointing device and calculating the matrix describing the linear transformation between the two (Eggers et al., 2006). The correspondence between imaging and the optical systems breaks down during the operation, however, due to a number of factors including cerebro-spinal fluid drainage, tissue resection, swelling of the brain, sagging under gravity, and the use of drugs such as osmotic diuretics (Peng et al., 2014). These factors contribute to displacements known as *brain shift*. This shift ranges between 5 and 10 mm (Hill et al., 1998; Roberts et al., 1998) and makes it increasingly difficult to take advantage of MR imaging information as surgery proceeds.

Brain shift can be measured and corrected using intraoperative ultrasound (US) (Comeau et al., 2000), laser-range scanning (Miga et al., 2016), or intraoperative MR imaging. Each has its merits and disadvantages. US is quite readily available and fast, although it has shown to be somewhat inaccurate in assessing the tumor border during resection (Rygh et al., 2008). Optical methods need a line of sight to be maintained between the camera, the probe and the reference frame during the operation. Intraoperative MR imaging necessitates specialized operating theaters and equipment and is associated with prolonged operation time. Studies reporting improved safety and more radical resection in brain tumor surgery with interoperative MR underline the importance of having reliable imaging available.

An alternative to intraoperative MRI, for tissue close to the surface of the cortex, would be to use local markers that move together with the shifting brain. The mesh of superficial cortical veins offers an appealing reference system; veins are clearly visible to the surgeon and move and deform with the immediately underlying tissue. In addition to the possibility of using surface veins for neuronavigation, the anatomy, and physiology of the venous system is also of great interest to the neurosurgeon *per se*. Iatrogenic damage to the major dural sinuses, the deep cerebral veins and superficial veins such as the vein of Labbe' can lead to oedema, swelling, intracranial tension, and hemorrhagic infarcts (Sindou et al., 2005), for instance, while the presence of veins in the sylvian fissure may preclude surgical intervention to clip aneurysms via pterional craniotomy, as damage to these can lead to hemorrhagic infarction (Kaminogo et al., 2002).

Compared to the arterial system, the venous system is highly variable between subjects (Oka et al., 1985), making it particularly important that it is well-studied in each patient. Digital Subtraction Angiography of the venous system yields only planar images and requires intravenous administration of dye into the carotid arteries. Computer Tomographic Angiography can be used to visualize the veins as well as the arteries in three dimensions (Kaminogo et al., 2002), whereas contrast-enhanced Magnetic Resonance Angiography (MRA) provides quite poor contrast in the veins. Many of these angiography techniques require the administration of contrast agent, which may not be possible in patients with renal problems, and are dependent on the vessels being well-perfused. Very slow flow or occlusion may also lead to them being overlooked.

Susceptibility-Weighted Imaging (Reichenbach and Haacke, 2001; Haacke et al., 2004) uses the influence of deoxygenated hemoglobin on both the phase and the magnitude of ${\text{T}}_{2}^{*}$ weighted gradient-echo scans to depict venous vessels at high spatial resolution, without the need for the administration of exogenous contrast agent. The static blood oxygenation level dependent (or BOLD) effect gives rise to signal changes which extend well beyond the physical extent of vessels, making SWI capable of imaging veins which are far smaller than the voxel size and which are not visible on anatomical imaging sequences with the same resolution. Higher signal-to-noise ratio and enhanced susceptibility effects at higher field strength have led to the wider use of SWI in neuroradiology at high (3 T) and ultra-high field (7 T). In many centers, SWI already contributes to the presurgical planning process, providing a high resolution map of veins which may need to be preserved or cauterized in the course of the operation. SWI provides images with exquisite contrast between veins and brain parenchyma, but the visualization of veins on the surface of the brain has been, so far, relatively poor, partly because the image processing involved in generating SWI tends to cause artifacts on the brain's surface. Additionally, the signal from the veins, which can be viewed as approximately cylindrical susceptibility structures, is convolved with a dipole kernel distribution. This process leads to “blooming” around the vein and non-local field effects which hamper localization of the vessels. In the context of segmentation, the magnitude of the T2*-weighted signal is therefore more generally used, particularly at ultra-high field.

Vessel segmentation is a burgeoning field of image processing which is applied to a range of medical imaging modalities. The methods applied may be fully automated and based on relatively simple vessel features such as their cylindrical shape or may incorporate multiple image properties in Gaussian mixture models and Markov random fields (Bazin et al., 2015; Ward et al., 2015). Classifier approaches, such as Random Forest, rely on a number of features and training data, usually manual vessel annotations (Rechberger et al., 2017). In this study we use what is probably the most widely established and tested basis for vessel segmentation in this context, the Frangi vesselness technique, which uses the eigenvectors of the Hessian matrix to isolate structures likely to correspond to vessels or other image ridges (Frangi et al., 1998).

The primary objective of our study was to develop and validate a non-invasive MRI technique for the visualization of the cerebral surface veins. The imaging is based on ultra-high field T2^*^-weighted imaging with automated vessel segmentation and overlay on anatomical MRI. The acuity of vessel overlays was assessed by reference to high resolution photographs in a study of human cadavers. Vessel segmentation was also performed in two healthy human subjects at both 7 and 3 T, allowing the feasibility of conducting surface vessel mapping *in vivo* on scanners at routine clinical field strength to be assessed.

## Methods

Two human cadaver heads were used in the study and two healthy male subjects, aged 27 and 38 years old, participated. This study was carried out in accordance with the recommendations of Ethics Committee of the Medical University of Vienna, which also approved the study protocol. All subjects gave written informed consent in accordance with the Declaration of Helsinki.

The authors had no information about the medical history of the cadavers. Measurements were made with a 7 Tesla MR whole body MAGNETOM scanner (Siemens Healthcare, Erlangen, Germany) and a 32 channel head coil (Nova Medical, Wilmington, USA). For the cadavers, the high resolution gradient-echo scan to be reconstructed was a 3D flow-compensated acquisition with 0.3 × 0.3 × 1.2 mm^3^ resolution, TE/TR = 10/28 ms, parallel imaging factor = 2; flip angle = 15°, acquisition time = 12 mins. The echo time used was decreased from that typically used *in vivo* (Deistung et al., 2008; Haacke et al., 2015; Springer et al., 2016), due to the additional signal dephasing encountered post mortem. Additionally, in the first cadaver scanned (Cadaver 1), T_1_-weighted data were acquired using an MPRAGE sequence with the following parameters: image-matrix = 320 × 320; resolution = 0.75 × 0.72 × 0.7 mm; number of slices = 208; parallel imaging factor = 2; T_R_/T_I_/T_E_ 3800/1700/3.55 ms; acquisition time = 10:29 min. No MPRAGE was acquired for Cadaver 2 for technical reasons. For the *in-vivo* subjects at 7 T the T2^*^-weighted imaging parameters were the same as for the cadavers, other than TE = 15 ms. At 3 T the resolution was 0.8 × 0.8 × 1.2 mm^3^ resolution, TE/TR = 28/36 ms, acquisition time 8 min 40 s.

Only magnitude images were used from the T2^*^-weighted acquisitions. To achieve robust and consistent vessel extraction, the following pre-processing steps were performed. All data were converted to the Medical Image NetCDF (MINC) format. As 7 T images tend to show low frequency intensity inhomogeneities arising from Radio Frequency field inhomogeneities, intensity correction plays a key role in successful vessel extraction. Intensity correction of the magnitude data was carried out using nu_correct (Sled et al., 1998), from the MINC-Toolbox. Afterwards, the image intensity from all subjects was rescaled to an arbitrary range (0–100).

Vessel segmentation was performed as a fully automated process, using a multi-scale method that uses second-order image information, represented by the Hessian matrix. The eigenvalues (λ_j_) of the Hessian matrix, sorted by increasing magnitude (|λ_1_| < | λ_2_| < | λ_3_|), describe the local second-order structure in an image. An example texture with an intensity gradient in two directions, e.g., in y (λ_2_) and z (λ_3_) direction and no intensity gradient in x (λ_1_) would be indicative of a structure pointing in the x direction (possible vessel) (Grabner et al., 2013). This method determines therefore the likeliness of a voxel to be part of a tubular structure (which is likely to be a vessel). As this approach works with a user-defined scale it is necessary to optimize segmentation parameters to detect only the vessels of the size of interest. The segmentation parameters α (which suppresses blob-like structures), β (which suppresses plate-like structures), and C (which thresholds the separation between noise and vessels) determine the sensitivity of the vesselness filter and the range of the spatial scale is defined by σ, which is the standard deviation of the Gaussian used to calculate the derivatives.

Here we used 0.5 as value for α and β, as suggested in Ref (Frangi et al., 1998; Manniesing and Niessen, 2005), and 1,500 for *C*. The spatial scale range was set between 0.5 and 3. A more detailed description of the chosen filter can be found in Refs (Frangi et al., 1998; Koopmans et al., 2008; Grabner et al., 2013) and a MATLAB version is available online (http:de.mathworks.com/matlabcentral/fileexchange/24409-hessian-based-frangi-vesselness-filter). For subject data with available T_1_ data (Cadaver 1, Subject 1 and Subject 2) automatic brain segmentation was performed using “mincbeast” (Eskildsen et al., 2012) with T_1_ data prior to 3D visualization. “mincbeast” uses a reference model with an accurate brain mask for segmentation. In order to cover the brain and surface veins this brain mask was manually dilated in the model space. The automatically created brain masks were then used to mask the segmented vessels in the individual data sets in order to include superficial veins. The brain surfaces were created using the “FreeSurfer” image analysis suite, which is documented and freely available for download online (http://surfer.nmr.mgh.harvard.edu/) (Dale et al., 1999; Fischl and Dale, 2000). The masked and segmented veins and the brain surface (pial surface) were for each subject 3D visualized using 3D Slicer (www.slicer.org) (Fedorov et al., 2012). 3D visualization of surface veins on Cadaver 2 was performed using a manually created brain mask, based on the magnitude data, and the Medical Imaging Interaction Toolkit (www.mitk.org) (Wolf et al., 2005).

### Details of craniotomy

Cadaver heads were stored at 4°C between MR measurements and removal of the brain, which was performed within 12 h. Prior to the craniotomy, the cervical part of the carotid artery and the jugular vein were dissected on both sides and ligated to reduce blood loss after dislocating the head from the corpus. The dural sack and both vertebral arteries between the 5 and 6th cervical vertebrae were likewise ligated to the same end. A circumferential skin incision was made with a scalpel 2 cm above both eye sockets, 2 cm above the external acoustic meatus on both sides and 1 cm above the occipital protuberance. The skin, underlying muscle, and fascia were removed en-bloc from the skull. The craniotomy was performed with an electric oscillating saw. The landmarks used for the skin incision served as orientation for the craniotomy. The calvaria was removed during constant blunt separation of the outer dura layer and the skull bone. After removing the skullcap, the dura was incised and cut away with scissors at the height of the craniotomy and removed en-bloc. The brain tissue was gently lifted, cranial nerves cut and the cerebellar tentorium incised in proximity to its insertion on the petrosal part of the temporal bone. Further lifting and dissection of the cranial nerves in the posterior cranial fossa allowed the brain to be removed from the neurocranium.

Photographic images of the cadaver brains were obtained with a 40 megapixel digital camera (Phase One, Copenhagen, Denmark) and a Schneider Kreuznach lens (Jos. Schneider Optische Werke GmbH, Bad Kreuznach, Germany) of 80 mm focal length, with a digital ISO of 100, f-number of 20, and 1/80 s exposure time. A very small light source was used to minimize reflections from the reflective and curved surface of the brain: a Hensel Porty strobe light with a EH Mini P LED Speed flash head (Hensel-Visit GmbH & Co. KG, Würzburg, Germany) and flood reflector.

Veins were identified on photographs and compared with their appearance on surface renderings by TH, a medicine graduate with anatomy and neurosurgery experience.

## Results

Segmentation and visualization was most successful for the parietal and superior anastomotic veins which could be identified in their entirety in both MR and photographs. The frontal, occipital, petrosal, temporal, and superior cerebral veins could be identified with interruptions. Not identified were inferior anastomotic, superficial middle, and cerebellar veins.

**Figure 1:**
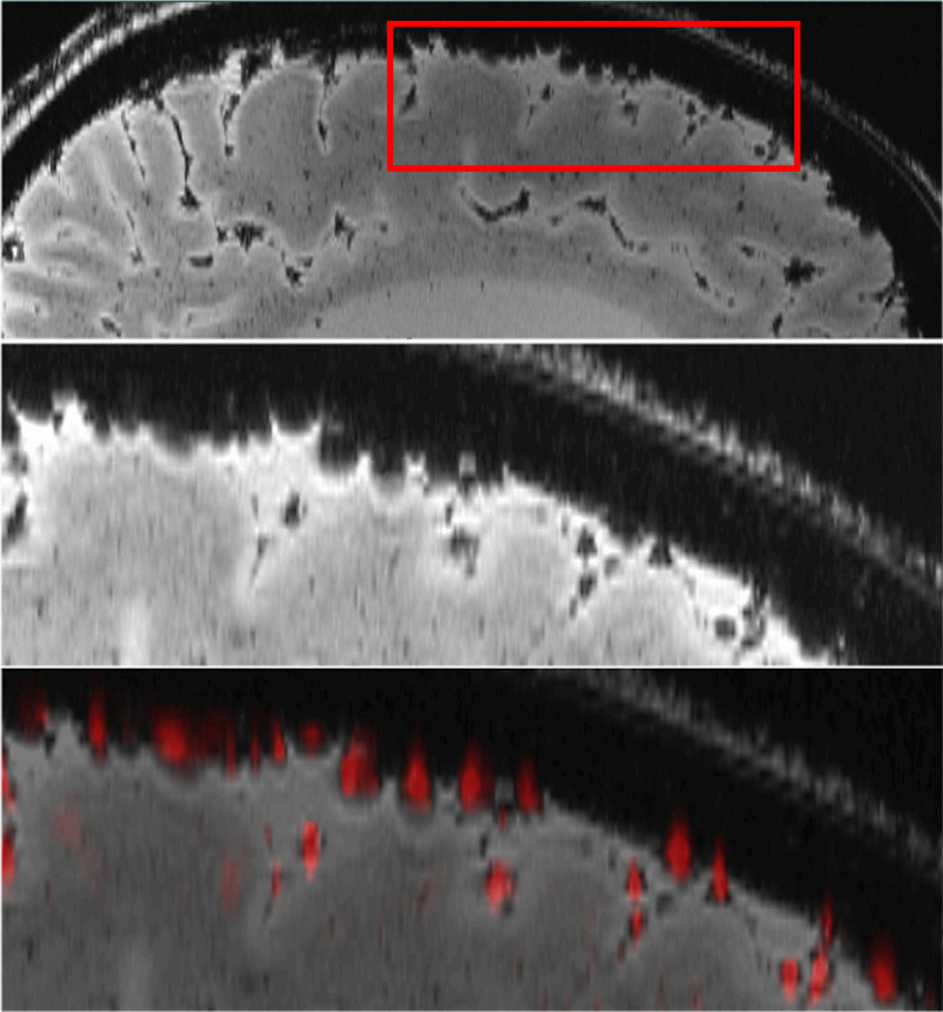
Automatic vessel segmentation: The top row shows a sagittal brain slice (magnitude of the T2^*^-weighted acquisition). An enlargement of the area within the red rectangle in the top row is shown in the middle row, where surface veins can be seen as hypointense, round structures. The bottom row shows the overlay of the automatically segmented surface veins (red) with the original magnitude image.

Figure 1 shows a sagittal brain slice (top and middle row; magnitude data from the T2^*^-weighted acquisition) of Cadaver 1, in which signal decay at the surface can be seen, such that surface veins are not surrounded by signal but are represented as round, hypointense structures reaching to half of their diameter into the cortex. The segmented vessels are shown in the bottom row of Figure 1 (overlaid on the magnitude data). It can be seen that hypointense structures are automatically segmented by red blobs representing the automatically segmented veins.

**Figure 2:**
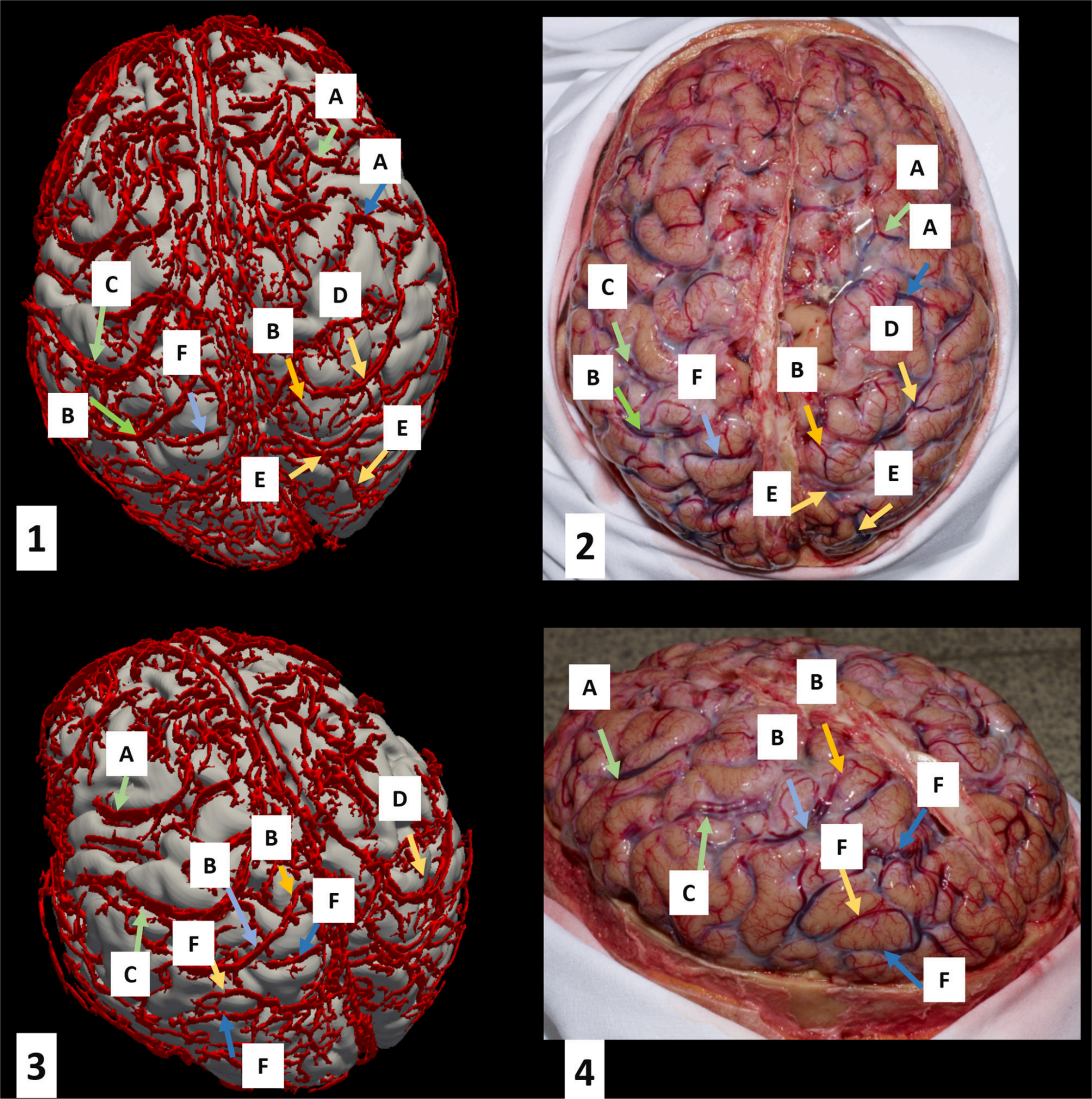
Superficial vein segmentation results for Cadaver 1 overlaid on T_1_-weighted images. The left column (quadrants 1 and 3) shows the 3D reconstruction of the segmented veins overlaid on the brain surface. The corresponding photographs are shown in quadrants 2 and 4. Vein labels on the left correspond to those of the right in the same row. The key is as follows: A, frontal veins; B, parietal veins; C, left superior anastomotic vein (of Trolard); D, right superior anastomotic vein (of Trolard); E, right occipital veins; F, left occipital veins.

**Figure 3:**
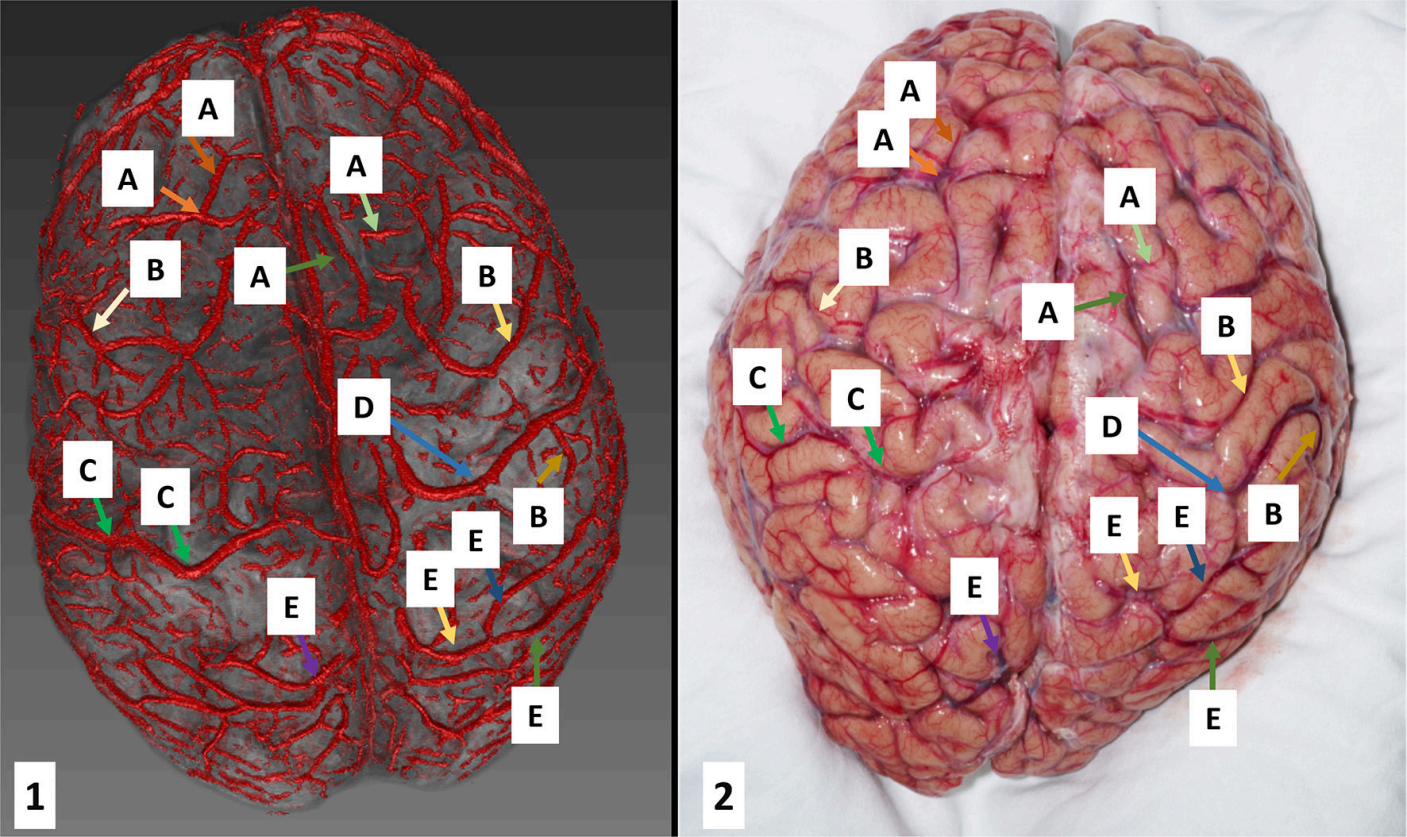
Superficial vein segmentation results for Cadaver 2 overlaid on anatomical data (left: the 3D reconstruction and right: the corresponding photograph). The characters represent: A, frontal veins; B, parietal veins; C, left superior anastomotic vein (of Trolard); D, right superior anastomotic vein (of Trolard); E, occipital veins.

**Figure 4:**
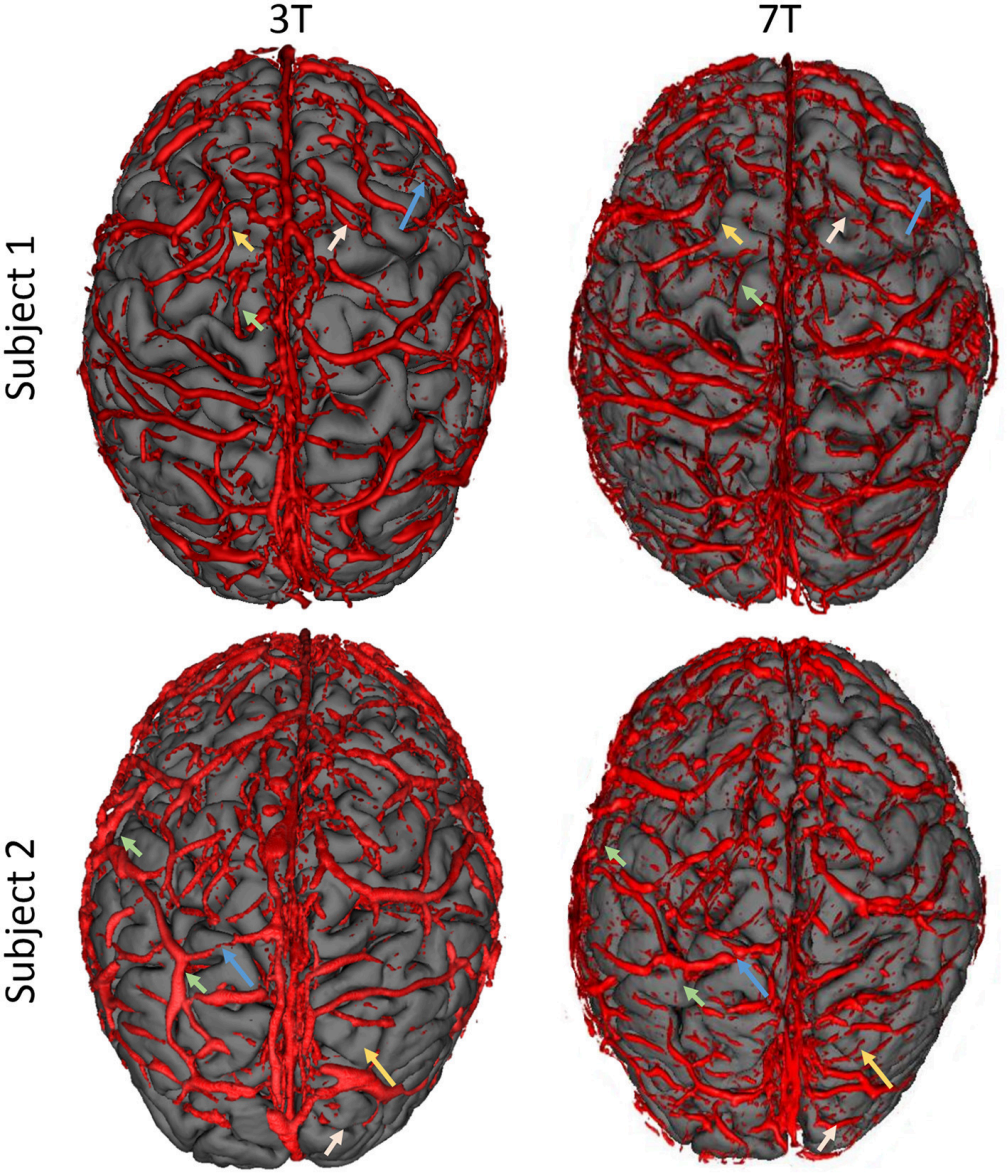
Comparison of visualized superficial veins from two control subjects (top and bottom row) at two different field strengths (3 T, left column and 7 T, right column). Overall, there is a high agreement of segmented veins between the two field strengths but there is also a certain mismatch. Some vessels could be visualized with 3 T which are not visible in 7 T results (short arrows) and vice versa (long arrows). Please note the limited reconstruction of the sagittal sinus, which is due to the segmentation parameters (which were selected for smaller vessels).

Figure 2 shows the segmented superficial veins in the area of the frontal, parietal, and occipital lobe of Cadaver 1 overlaid on T_1_-weighted data. The superior anastomotic veins of Trolard (Figure 2; C and D), which connect the superior sagittal sinus and the superficial middle cerebral vein, are clearly visualized and traceable on the 3D reconstructions (Figure 2; 1 and 3) as well as on the photographs (Figure 2; 2 and 4). Additionally, superior cerebral veins, which drain the superior portion of the cerebral cortex, are clearly visualized on the 3D reconstruction (Figure 2; 1 and 3) as well as on the photographs (Figure 2; 2 and 4). A large portion of the superior cerebral veins can be traced up and medially before they drain into the superior sagittal sinus. The sagittal sinus does not appear clearly in the 3D segmentation because its size was not covered by the segmentation parameters. Due to increasing segmentation artifacts with adapted segmentation parameters a limited 3D reconstruction of the sagittal sinus was accepted. Figure 2 also shows examples of selected occipital veins (E and F).

Figure 3 illustrates segmentation results for Cadaver 2 overlaid on the magnitude data of the same scan. As can be seen, veins were cleanly segmented from the frontal to the occipital lobes. There was more deformation of the brain between the MR measurement and the photograph for Cadaver 2, because, in contrast to Cadaver 1, which was photographed still in the skull, this brain was removed completely. Complete removal of the brain led to deformation which was larger than would usually be encountered in surgery but nonetheless illustrates how problematic it is, in the absence of surface landmarks, to identify tissue within a brain which has lost correspondence to prior skull-based imaging.

Figure 4 shows the rendered surface veins of the two control subjects at both field strengths (3 T and 7 T). As can be seen, there is a certain mismatch between the two field strengths but major vessels are visible in both subjects at both field strengths.

## Discussion

Pre-operative multimodal planning of neurosurgical procedures drastically reduces morbidity and mortality by reducing collateral damage to eloquent areas and adjacent neurovascular structures. Meticulously acquired and co-registered pre-operative data loses accuracy during surgery due to brain shift after craniotomy. Intraoperative MR imaging provides a possible improvement but is, due to the expensive infrastructure required, not available in all neurosurgical departments. Precise information about the superficial cerebral veins have the potential to form a network of reference points which retain their relevance throughout surgery.

Here, we used a non-invasive contrast-agent-free MR imaging technique (a gradient-echo-based sequence) and an image post-processing technique that relies on a Hessian matrix-based vesselness filter to visualize veins on the brain's surface. These veins were then confirmed by autopsy, which is an essential step toward the utilization of surface venograms as landmarks for neurosurgery. Having a reliable map of the superficial veins is not only of use in localization and landmarks for neurosurgery; it may also assist in surgical planning. The presence of surface veins may restrict access to malformations and the removal of too many draining veins can lead to postoperative edema (Sindou et al., 2005). The visualization of cortical venous thrombosis and study of the variability of the venous network across subjects are possible further applications.

A mesh of superficial cortical veins as generated in this study could be combined with morphological, fMRI, DTI and other MRI information, all of which are in a skull-based Euclidean space. The veins, which are clearly visible to the neurosurgeon, could provide a reference framework for images as the brain deforms. The clinical utility of this method has yet to be established, but it is expected to be primarily applicable for tissue close to the surface.

For optimal visualization, segmented veins should be overlaid on T_1_-weighted images because of the higher anatomical contrast of these scans, and the better depiction of sulci and gyri. For instance, Figure 3 represents 3D reconstructed veins overlaid to the magnitude data. As there is less tissue contrast (anatomical information) in the magnitude data (see Figure 1) the patterns of cortical folding cannot be visualized as well as when using T_1_ weighted data (see Figure 2).

The vessel identification algorithm used in this study was applied to magnitude images rather than to phase images, SWI, QSM, or “true SWI” (vessel contrast derived from QSM, rather than filtered phase; Haacke et al., 2015). Although phase is an important contributor to vessel contrast in SWI, most other segmentation studies have also used the magnitude signal only (Koopmans et al., 2008). There are a number of reasons for this. First, the dipole distribution by which the susceptibility is convolved is larger at 7 T, meaning that veins are visualized much bigger than their true size (the “blooming effect”). This tends to modify the shape of veins at the brain's surface from cylinders to channels, or grooves. This is exacerbated at the surface by the high pass filtering of the raw phase images (Rauscher et al., 2008). Both of these effects result in poor recognition of vessels and over-segmentation at the surface. Secondly, while deoxyhemoglobin concentration leads to local magnitude signal decay, phase effects are both non-local and orientation dependent (see e.g., Ref Schweser et al., 2015), which can lead to the appearance of two veins, on either side of the true location of the vessel, depending on the angle the vein makes to the static magnetic field. This has been overcome in recent work using QSM or true-SWI rather than SWI, which have the potential to remove non-local and orientation-dependent problems encountered using the phase. In practice, though, most background field correction methods used for QSM require an erosion of the brain volume to generate a ROI for background field correction (Schweser et al., 2017), in which process surface veins get lost (Bazin et al., 2015). For that reason (and others, such as noise amplification), vessel segmentation results based on QSM have been shown to relate more poorly to ground truth vessels than those based on SWI, even in an assessment performed over a range of vessels (rather than just surface vessels) and at 3 T (rather than at 7 T where brain segmentation is more problematic and signal loss is exacerbated at the surface) (Ward et al., 2015).

The data acquired here for verification of vessel identification were obtained from cadavers in which the oxygen saturation is lower than it is *in-vivo* and where the degeneration of hemoglobin leads to an increase in the magnetic susceptibility of blood, and an associated increase in susceptibility effects. Bigger veins and over-segmentation result. In this study, over-segmentation was reduced by using a shorter TE for data acquisition (10 ms) compared to that typical for *in-vivo* measurements (Deistung et al., 2008; Robinson et al., 2011, 2017; Grabner et al., 2012) (and the *in-vivo* measurements here) and using magnitude data for segmentation. The segmentation of superficial veins in living subjects is, in many regards, less problematic.

The expansion of the region of signal dephasing outside the vessel has the advantage that vessels which are smaller than the voxels may be visualized. A disadvantage is that there is no direct relation between the size of the vein in segmented images and their true size. Corresponding veins can be identified (see, e.g., the figures presented here) but this effect could add some uncertainty in determining the position of the periphery of a vessel, which could be important in electrode placement, for instance. Post-processing techniques are being developed to better estimate the true vessel size (Ward et al., 2017).

In general, the use of gradient-echo based ${\text{T}}_{2}^{*}$ imaging has some advantages over techniques based on the administration of an external contrast agent. First, SWI is exquisitely sensitive to small venous vessels. Second, not having to use a contrast agent means that the contrast agent administration can be used e.g., for perfusion imaging. Finally, many patients have contraindications to contrast agent administration such as severe renal insufficiency with potential development of a nephrogenic systemic fibrosis (Dill, 2008)—a rare but very serious condition (Lauenstein et al., 2007; Gossner, 2009). The use of gradient-echo based T2^*^ imaging, rather than contrast-enhanced imaging is in line with the general trend toward reducing or avoiding contrast agent administration wherever possible, particularly in children.

In this study we used ultra-high field (7 T) MRI, which has a higher signal to noise and BOLD contrast than the 1.5 and 3 T MRI scanners in current clinical use. While this was a contributory factor in being able to generate high resolution images with high SNR, the rapid signal dephasing of signal on the surface of the brain also make vein recognition more problematic there. Indeed, *in vivo* results demonstrated that, for the larger veins on the cortical surface, the quality of 3 T segmentation results was similar to that of the 7 T results. This is consistent with previous 3 T findings demonstrating impressive maps of surface vessels (Al-Rekabi et al., 2009; Bériault et al., 2015) although the correspondence of those maps to the real vasculature could not be verified in that prior work. Intracranial veins are also routinely imaged at 1.5 T using SWI making translation to field strengths below 3 T a worthwhile goal. This may be achieved in the future with improved coil technology or improved processing of the phase information, e.g., if improved brain segmentation and background field removal allows “true SWI” be used to remove the non-local effects and orientation dependence, even at the brain's surface. A further limitation of this study is that only a small number of brains were investigated. This was due to the fact that imaging was not possible in a number of candidate cadavers because of gross signal dropouts caused by the ingression of air and because of the complexity of the experimental procedure, comprising 7 T MRI, excision and photography. However, even with a limited number of subjects, we were able to show that the surface veins can be mapped with MRI and that the imaging data correspond to the actual venous anatomy. Further work, involving a comparison of *in-vivo* imaging of patients scheduled for surgery and intraoperative photographs, is required in order to assess the clinical potential of this approach and its practical utility as a means of dealing with brain shift.

## Conclusions

The correlation between automatically generated surface venograms created using a non-invasive, contrast-agent-free gradient-echo imaging method and high resolution autopsy photographs indicates that surface venograms are sufficiently complete and reliable to be a candidate landmark system for neurosurgery. The feasibility of this method needs to be established in a larger *in-vivo* study at clinical field strength in neurosurgical cases.

## Ethics statement

Ethics Committee Vienna 1561/2014 (7T fMRI).

## Author contributions

GG and AR: Writing, image processing. TH: Cadaver preparation, writing, medical contribution. MG: Photographer, image processing. HT: Cadaver preparation, medical contribution. ST: Medical contribution, MR imaging. SR: Writing, image processing, MR imaging.

### Conflict of interest statement

The authors declare that the research was conducted in the absence of any commercial or financial relationships that could be construed as a potential conflict of interest.
